# Hemolysis, elevated liver enzymes, and low platelet (HELLP) syndrome in a 26-year-old woman with cystic fibrosis: a case report

**DOI:** 10.1186/1752-1947-6-134

**Published:** 2012-05-23

**Authors:** James Geake, Eli Dabscheck, David Reid

**Affiliations:** 1 Department of Respiratory and Sleep Medicine, Monash Medical Centre, 246 Clayton Road, Clayton, 3168, Australia; 2 Department of Respiratory Medicine, Hobart, 7001, Tasmania, Australia; 3 Department of Allergy, Immunology and Respiratory Medicine, Alfred Hospital, Commercial Road, Prahan, Melbourne, 3181, Australia; 4 Adult Cystic Fibrosis Centre, The Prince Charles Hospital, Rode Road, Chermside, Queensland, 4032, Australia; 5 Tasmanian Adult Cystic Fibrosis Unit, Hobart, 7001, Tasmania, Australia

## Abstract

**Introduction:**

The microangiopathic hemolysis, elevated liver enzymes, and low platelet (HELLP) syndrome is a rare and potentially fatal complication of pregnancy. Here, to the best of our knowledge, we describe only the second reported case of this syndrome in a woman with cystic fibrosis.

**Case presentation:**

This report describes the case of a 26-year-old woman of Caucasian ethnicity with mild cystic fibrosis bronchiectasis who ultimately manifested the triad of microangiopathic hemolysis, elevated liver enzymes, and low platelet count in the third trimester of pregnancy. Her baby was delivered successfully after a semi-elective caesarean section.

**Conclusions:**

Pregnancies in patients with cystic fibrosis are associated with an increased rate of complications. This case is of importance as it describes only for the second time the successful delivery of a baby in a women with cystic fibrosis, in a pregnancy also threatened by the microangiopathic hemolysis, elevated liver enzymes, and low platelet syndrome. This case will be of special interest to obstetricians, pediatricians, and medical, nursing and allied health staff involved in the delivery of cystic fibrosis care.

## Introduction

‘Hemolysis, Elevated Liver enzymes, and Low Platelet count’ (HELLP) syndrome is a rare complication of pregnancy. It usually occurs in the setting of severe pre-eclampsia and manifests almost exclusively after the 20th week of gestation, with up to 30% of cases occurring in the immediate post-partum period [1]. It can be life-threatening to both mother and baby, and definitive treatment is delivery of the baby, which usually results in resolution [2]. Here, we describe only the second reported case of a young woman with mild cystic fibrosis (CF)-related lung disease who presented in the third trimester of her second pregnancy with HELLP syndrome [3].

## Case presentation

A 26-year-old Caucasian woman who had mild lung disease presented to our facility. Her pre-partum forced expiratory volume in one second (FEV) was 2.62 L (87% predicted). Her pre-partum body mass index (BMI) was 24. She was chronically infected with *Pseudomonas aeruginosa*. In addition, she was pancreatically insufficient and had CF-related diabetes treated with insulin. Her glycemic control was acceptable with a hemoglobin A1C (HbA1C) level of 6.1%. She did not have microalbuminuria as estimated by a spot albumin:creatinine ratio. Eight years previously she had delivered a healthy child via a normal vaginal delivery after spontaneous rupture of membranes at 36 weeks’ gestation. The pregnancy had been uncomplicated.

Her second pregnancy was unplanned and she had conceived with a new partner. Following confirmation of the pregnancy, she was referred to a high-risk pregnancy clinic where she was reviewed regularly by a multidisciplinary team supervised by an obstetrician, a respiratory physician and an endocrinologist. The first two trimesters proceeded without significant medical complication. During the 26th week of pregnancy, a routine out-patient review revealed a fall in her lung function (FEV_1_ 2.13 L, approximately 71% predicted) accompanied by increased respiratory symptoms. Her blood pressure was noted to be slightly elevated at 118/87. A hospital admission for treatment of her fall in lung function was declined and she was given a two-week course of oral ciprofloxacin at a dose of 750 mg twice a day. This was tolerated poorly due to nausea.

Two weeks later, in the 28th week of pregnancy, she developed worsening nausea and further exacerbation of her respiratory symptoms with some accompanying left upper quadrant discomfort. Her blood pressure was 123/90. The results of a urinary test strip screening for proteinuria were negative. A hospital admission was again declined and treatment with oral ciprofloxacin for a presumed mild pulmonary exacerbation was continued, and she was prescribed oral metoclopramide, with outpatient follow up arranged in one week. She was advised to present to hospital sooner in the event of clinical deterioration.

Our patient re-presented one week later with increasing nausea and a persistent impairment of lung function (FEV_1_ 2.09; 69% predicted). She was admitted to hospital. At this time, her blood pressure was 153/89. Laboratory investigation results revealed hemoglobin of 12.3 g/dL, platelet count of 101 × 10^9^ cells/ml (normal range 150 to 400 × 10^9^ cells/ml), serum alanine transaminase (ALT) of 179 mmol/L (normal range <65 mmol/L), lactate dehydrogenase (LDH) of 279 mmol/L (normal range <240 mmol/L) and urate of 523 mmol/L (normal range 150 to 400 mmol/L). There was no significant proteinuria as assessed by a urinary protein/creatinine ratio and this remained unchanged throughout the admission. Similarly, serial assessment of her coagulation and renal function failed to reveal any abnormality. On admission, a fetal ultrasound was within normal limits.

A diagnosis of pre-eclampsia with some features of HELLP syndrome was made, in the setting of a respiratory exacerbation. In addition to her regular medications, she was put on intravenous aztreonam at a dose of 2 g every six hours. She was given two 11.4 mg doses of intramuscular betamethasone at 12-hour intervals to assist with fetal lung maturation. Aminoglycosides were not given to avoid any potential harm to the fetal kidneys. Over the next five days she remained hypertensive with a systolic blood pressure ranging from 150 to 190 mmHg, but her platelet count increased to 239 × 10^9^ cells/ml and the results of liver function tests (LFTs) progressively normalized. However, on the fifth day there was a precipitous drop in platelet count to 62 × 10^9^ cells/ml and an increase in ALT to 118 mmol/L, accompanied by a fall in hemoglobin to 8.8 g/dL with red cell fragments observed on blood films taken at this time. A diagnosis of HELLP syndrome was made and a semi-elective caesarean section was performed 10 hours later under spinal anesthesia. HELLP-specific treatment, in particular magnesium sulfate was not administered. A healthy baby girl was delivered. Her APGAR scores were 9 and 9 at one and five minutes, respectively. All of our patient’s blood parameters normalized the day following birth of her child. Our patient recovered without further medical complication. Four years later, our patient’s lung function remains stable, although she continues to have intermittent exacerbations of her bronchiectasis. Our patient’s child has reached normal age-specific milestones.

## Discussion

HELLP syndrome complicates approximately one to two in every 1000 pregnancies. Many consider it a severe variant of the pre-eclamptic spectrum, although this is controversial as at times it occurs without other pathological hallmarks of the disease, as occurred in this case with the absence of proteinuria. Although the pathophysiology of pre-eclampsia is yet to be fully defined, broadly it is thought to arise as a consequence of deficient placentation, followed by the liberation of vasoactive mediators and subsequent maternal endothelial dysfunction [4]. Risk factors for the development of HELLP syndrome are: multiparity, age over 25 years, Caucasian ethnicity, and a history of poor pregnancy outcome [5]. The definitive treatment for both pre-eclampsia and HELLP syndrome is delivery of the baby, and there is broad consensus that this should be expedited if the pregnancy is at or beyond 34 weeks of gestation and especially if assessments of fetal status are not reassuring, or if there is significant maternal disease as in this case. While the presence of cystic fibrosis and HELLP syndrome may not have been of direct relevance to each other in this case, the diagnosis of HELLP syndrome was somewhat obscured. Individuals with CF frequently develop abnormal LFTs when unwell or dehydrated because of biliary sludging and she appeared to be making a recovery before acutely deteriorating, at which point the diagnosis became clear.

With respect to CF lung disease and pregnancy, small case series have not documented adverse maternal outcomes for patients with relatively preserved lung function (FEV_1_ > 60%), although rates of prematurity would appear to be increased [6]. It is unlikely that the manifestation of HELLP syndrome adversely impacted on our patient’s lung function. However, although the outcome was favorable in the case of our patient, our patient’s CF added complexity to acute management as her lung function had deteriorated and people with CF are more at risk during general anesthetic procedures, especially if a peri-operative inability to clear airway secretions is compounded by post-operative abdominal pain.

The major differential diagnoses to consider in this case were thrombotic thrombocytopenic purpura and the hemolytic uremic syndrome. However, the presence of hypertension prior to the development of severe microangiopathic hemolysis and platelet destruction, as well as the absence of any associated renal dysfunction, plus the rapid resolution of clinical and biochemical derangements upon delivery of the baby argues against these other diagnoses.

## Conclusions

To the best of our knowledge this is only the second reported case of HELLP syndrome manifesting in a pregnant individual with CF. The case highlights the importance of delivery of the baby as the definitive management route for the disorder. As survival in CF increases and an increasing number of women are becoming mothers, there needs to be a low threshold of suspicion when patients develop a slight worsening of their LFTs in the setting of an elevated BP and the findings in our case caution against attributing some of the biochemical manifestations of HELLP syndrome simply to the underlying CF. Furthermore it demonstrates the importance of managing CF pregnancies through multidisciplinary high-risk antenatal clinics.

## Consent

Written informed consent was obtained from the patient for publication of this case report and any accompanying images. A copy of the written consent is available for review by the Editor-in-Chief of this journal.

## Competing interests

The authors declare that they have no competing interests.

## Authors’ contributions

JG, ED, and DR reviewed the case and completed the case summary. JG and DR reviewed the literature and completed the case discussion. All authors read an approved the final manuscript.
